# *Tis21*-gene therapy inhibits medulloblastoma growth in a murine allograft model

**DOI:** 10.1371/journal.pone.0194206

**Published:** 2018-03-14

**Authors:** Dario Presutti, Manuela Ceccarelli, Laura Micheli, Giuliana Papoff, Simonetta Santini, Simone Samperna, Cristiana Lalli, Lorena Zentilin, Giovina Ruberti, Felice Tirone

**Affiliations:** 1 Institute of Cell Biology and Neurobiology, National Research Council (IBCN-CNR), Monterotondo, Rome, Italy; 2 Institute of Cell Biology and Neurobiology, National Research Council (IBCN-CNR), Fondazione Santa Lucia, Rome, Italy; 3 International Centre for Genetic Engineering and Biotechnology (ICGEB), Padriciano, Trieste, Italy; Universidad de Navarra, SPAIN

## Abstract

Medulloblastoma (MB), the tumor of the cerebellum, is the most frequent brain cancer in childhood and a major cause of pediatric mortality. Based on gene profiling, four MB subgroups have been identified, i.e., Wnt or Sonic Hedgehog (Shh) types, and subgroup 3 or 4. The Shh-type MB has been shown to arise from the cerebellar precursors of granule neurons (GCPs), where a hyperactivation of the Shh pathway leads to their neoplastic transformation. We have previously shown that the gene *Tis21* (*PC3*/*Btg2*) inhibits the proliferation and promotes the differentiation and migration of GCPs. Moreover, the overexpression or the deletion of *Tis21* in *Patched1* heterozygous mice, a model of spontaneous Shh-type MB, highly reduces or increases, respectively, the frequency of MB. Here we tested whether Tis21 can inhibit MB allografts. Athymic nude mice were subcutaneously grafted with MB cells explanted from *Patched1* heterozygous mice. MB allografts were then injected with adeno-associated viruses either carrying *Tis21* (AAV-*Tis21*) or empty (AAV-CBA). We observed that the treatment with AAV-*Tis21* significantly inhibited the growth of tumor nodules, as judged by their volume, and reduced the number of proliferating tumor cells (labeled with Ki67 or BrdU), relative to AAV-CBA-treated control mice. In parallel, AAV-*Tis21* increased significantly tumor cells labeled with early and late neural differentiation markers. Overall the results suggest that *Tis21*-gene therapy slows down MB tumor growth through inhibition of proliferation and enhancement of neural differentiation. These results validate *Tis21* as a relevant target for MB therapy.

## Introduction

Medulloblastoma (MB), a highly malignant cerebellar neoplasm, is the most common brain cancer in infants and children, comprising 15–20% of all pediatric nervous system tumors. Moreover, MB represents the primary cause of pediatric mortality related to cancer. MB is also seen in adults, but it only accounts for 1.0% of adult brain tumors [1–5]. Currently, patients undergo surgical resection, chemotherapy and craniospinal irradiation, with devastating late and long-term side effects [4–8]. Thus, the experimental research is now directed to develop molecular therapies, aimed to increase the specificity for cancer cells and minimize the damage to the developing brain.

Based on the molecular profiling, the MBs can be classified into four molecular subgroups: Wnt, Sonic hedgehog (Shh), subgroup 3 and subgroup 4 [9, 10].

The Shh subgroup comprises approximately one-third of all cases of human MBs [11, 12]; moreover, to this group belongs the large majority of published MB animal models (e.g., the mice heterozygous for *Patched1*, a Shh receptor inhibiting the Shh pathway) [11, 13–16].

The Shh-dependent MBs originate from constitutive activation of the Shh signal pathway, which controls the proliferation of cerebellar granule precursor cells (GCPs) in the external granular layer (EGL) during the physiological cerebellar maturation, before their migration and differentiation in granule neurons in the internal granular layer (IGL) [17, 18]. The hyperactivation of the Shh pathway causes the multiplication of GCPs at the cerebellar surface and promotes the cell transformation, with consequent formation of neoplastic GCPs [19–26]. Thus, the recent MB therapeutic approaches have employed Shh inhibitors, but unfortunately resistance to these small molecules has been reported [12, 27–31].

The *Tis21* gene (also known as *PC3* or *Btg2*, in rat and human [32]) encodes a transcriptional cofactor that functions as a tumor suppressor protein in prostate cancer cells [33], in renal cancer development [34] and in breast cancer [35]. Tis21 also plays an important role in the proper development of the cerebellum and, therefore, in the pathogenesis of MB. In the GCPs, Tis21 acts as a functional antagonist of Shh and modulator of cell proliferation by direct repression of the *cyclin D1* promoter with recruitment of HDAC1 and HDAC4 [36, 37]. Moreover, Tis21 induces GPCs terminal differentiation through the expression of the neural transcription factor *Math1* [36].

We have previously reported that overexpression of the *Tis21* gene in GCPs of *Patched1* heterozygous mice (*Ptch1*^+/-^), a model of spontaneous Shh-MB, is able to strongly reduce the incidence of MB and the number of preneoplastic lesions by inhibiting cell proliferation and triggering granule cells differentiation [37]. Moreover, we observed a down-regulation of *Tis21* expression in murine and human MBs of different isotypes [37].

Conversely, a dramatic increase in MB frequency occurs in *Ptch1*^+/-^ mice when crossed with mice knockout for the *Tis21* suppressor gene (*Ptch1*^+/-^*/Tis21*-/-), due to an impairment of GCPs migration from the cerebellar surface to the inner layers during the development of the cerebellum [38]. In fact, the deficit of migration restrains the GCPs for a longer period of time within the external proliferative zone where these cells, instead of differentiating and migrating internally, become prone to transformation [38–41]. As we revealed by genome-wide analysis, Tis21 affects the migration of GCPs through the direct transcriptional control of the *Cxcl3* chemokine [38, 42]. The treatment *in vivo* with Cxcl3 significantly reduces the development of preneoplastic lesions [38, 43]. Surprisingly, the *Tis21* deletion did not result in changes in the proliferation rate of GCPs in the EGL, likely for the action of the highly homologous family-related gene *Btg1* [44].

In this study, we analyzed the therapeutic potential of Tis21 by testing whether Tis21 virally transduced in MB allografts can inhibit their growth. To this aim, MB cells derived from *Ptch1*^+/-^ mice were grafted subcutaneously in nude mice and the developing tumor nodules were injected with an adeno-associated viral vector (AAV) able to efficiently transduce and express exogenous *Tis21* gene in GCPs and in cerebellar granule neurons. We observed that the treatment with the AAV-*Tis21* slows the growth of tumor nodules by reducing cell proliferation and promoting neural differentiation. Therefore, our results confirm the role of *Tis21* as a MB suppressor gene and validate *Tis21* as a potential relevant target for gene therapy of brain tumors.

## Materials and methods

### Cells, reagents and antibodies

The human medulloblastoma cell lines DAOY (ATCC® HTB-186™) and D283 (ATCC® HTB-185™) were cultured in MEM Eagle medium (BioWhittaker, Lonza, Walkersville, MD, USA) supplemented with Earle’s BSS, 2 mM L-glutamine, 100 U/ml penicillin/streptomycin, 1 mM sodium pyruvate, 0.1 mM non essential aminoacids (BioWittaker, Lonza) and heat inactivated 10% fetal bovine serum (Sigma-Aldrich, St. Louis, MO, USA). All cells were cultured at 37°C in a 5% CO_2_ humidified incubator.

Collagenase type IV, hyaluronidase and 5-bromo-2’-deoxyuridine (BrdU) were from Sigma-Aldrich. Corning matrigel basement membrane matrix growth factor reduced and cell-strainer (40 μm) were from BD Biosciences (San José, CA, USA). The primary antibodies: Ki67 rabbit monoclonal antibody (clone SP6; RM-9106-S1; 1:150) was from Thermo Fisher Scientific (Waltham, MA, USA); BrdU rat monoclonal IgG2a antibody (clone BU1/75; MCA2060; 1:300) was from AbD Serotec (Raleigh, NC, USA); NeuroD1 goat polyclonal antibody (AF2746; 1:200) was from R&D Systems (Minneapolis, MN, USA); NeuN mouse monoclonal IgG1 antibody (clone A60; MAB377; 1:300) was from Millipore (Temecula, CA, USA). Streptavidin Alexa Fluor-488 (S11223; 1:500) was from Thermo Fisher Scientific. The Cy3-conjugated streptavidin and the secondary antibodies used to visualize the markers in the free-floating sections (a donkey anti-rat Cy2-conjugated for the BrdU, a donkey anti-rabbit Cy2-conjugated for the Ki67 and a donkey anti-goat Cy3-conjugated for the NeuroD1) were all from Jackson ImmunoResearch (West Grove, PA, USA; 1:200). Biotin-labeled horse anti-mouse (BA-2000; 1:300), anti-rabbit (BA-1100; 1:300) and anti-goat (BA-9500; 1:300) IgG secondary antibodies were from Vector Laboratories (Burlingame, CA, USA). SYBR select Master Mix was purchased from Applied Biosystems (Foster City, CA, USA). Oligonucleotides were synthetized by Eurofins Genomics (Ebersberg, Germany).

### Ethics statement

Animals were subjected to experimental protocols (Authorizations N. 193/2015-PR and N. 872/2015-PR) approved by the Veterinary Department of the Italian Ministry of Health. Experiments were conducted according to the ethical and safety rules and guidelines for the use of animals in biomedical research provided by the relevant Italian law and European Union Directive (Italian Legislative Decree 26/2014 and 2010/63/EU) and the International Guiding Principles for Biomedical Research involving animals (Council for the International Organizations of Medical Sciences, Geneva, CH). All adequate measures were taken to minimize animal pain or discomfort.

### Adeno-associated Virus-*Tis21* expressing, cloning and infection

Packaging of plasmids in recombinant AAV serotype 9 (hereafter named AAV) is based on methods previously developed and described by Klein et al. [45].

Briefly, 293 helper cells have been transfected with an AAV terminal repeat containing plasmid (either pAAV-CBA-WPRE-empty or pAAV-CBA-WPRE-*GFP* used as control vectors or pAAV-CBA-WPRE-*Tis21*, abbreviated in AAV-CBA, AAV-*GFP* or AAV-*Tis21*, respectively). R. Klein kindly provided the *pGFP* plasmid, from which we excised the *GFP* insert contained in the HindIII site, obtaining AAV-CBA (that carried no insert), and then we cloned in the HindIII site of AAV-CBA the *Tis21* (mouse) open reading frame, thus generating AAV-*Tis21*. Transfection of AAV-CBA, AAV-*GFP* or AAV-*Tis21* occurred in an equimolar ratio with the plasmid pDG, which provides the AAV coat protein genes, and adenovirus 2 genes necessary for helper function in packaging. The plasmids have been packaged in recombinant AAV serotype 9 because this is able to infect CNS cells [46]. Three days after transfection, cells and media have been harvested, pelleted, resuspended in lysis buffer (50 mM Tris, pH 8.5, 150 mM NaCl) and freeze–thawed three times. The samples have been incubated with endonuclease (1500 units for 30 min at 37°C), centrifuged at 2000 x g and the resulting supernatants applied to a discontinuous gradient of cesium (Sigma-Aldrich), followed by dialysis against 1x PBS. Recombinant AAVs has then been titered for total particles by the described dotblot and quantitative/competitive PCR methods [45] and used for human MB cell lines infections and allograft treatments.

### Cell lines AAV infections

DAOY and D283 cells were seeded respectively at 50,000 and 150,000 cells/60 mm plate overnight. They were then infected with AAV (CBA or *Tis21*) at four different multiplicity of infection (MOI) ranging from 1.3x105 to 6.2x106 viral particles/cell in complete tissue culture medium. Seven days post infection, RNA was harvested using Trizol reagent (Thermo Fisher Scientific) and used to perform quantitative reverse-transcriptase PCR (qRT-PCR).

### MB development and isolation of tumor cells for allograft propagation

*Ptch1*^+/-^ mice were obtained in a CD1 background by gene targeting of exons 6 and 7 [47] and were maintained by continuous inbreeding. The pups were routinely genotyped by PCR, using genomic DNA from tail tips, as previously described [37, 47]. It is known that the *Ptch1*^+/-^ mice on the CD1 background develop spontaneous MB over a period of 10 months with low incidence (about 7%) [48]. Thus, *Ptch1*^+/-^ mice of either sex were daily observed for tumor formation for 12 months after birth. On the appearance of MB symptoms, such as weight loss, ruffling of fur, lethargy, poor balance, posterior paralysis and impaired coordination, they were euthanized and the MB tumors isolated. The tumor cell suspension was obtained from explanted MB following the protocol described by Sasai et al. [49]. Briefly, MB tumor was reduced in small fragments by mean of a McIlwain tissue chopper and incubated with collagenase type IV (500 μg/ml) and hyaluronidase (500 μg/ml) for 30 minutes at 37°C, then strained using a cell-strainer (40 μm). A yield of 2–4 x 107 cells per tumor was obtained.

### Allograft in nude mice and AAV-treatments

Athymic nude female mice (*Foxn1nu/Foxn1*+) (Envigo, Udine, Italy) were housed in individually ventilated cages under controlled conditions (20–22°C; 55–65% relative humidity; 12/12 hours light/dark cycle; irradiated standard diet and water *ad libitum*).

Tumor allografts were generated essentially as previously described [50]. Briefly, 5–10 weeks old mice were injected subcutaneously with MB cells (2.5–3.0 x 106 cells) in 50% matrigel in 200 μl of PBS into the dorsal flank region of each mouse. Tumor volume was calculated by caliper measurements of tumor length (L) and width (W) according to the formula: *L* × *W*2 ÷ 2.

Engrafted MB cells were allowed to expand before treatment with AAV. When tumors reached an approximate volume of 100–150 mm3 the mice were randomly divided into an experimental and control groups (n = 2-5/group) for AAV or PBS treatment. Six intratumoral injections (T1-T6) of AAV-*Tis21* or AAV-CBA or AAV-*GFP* (50 μl equivalent to 1011 viral particles), or PBS (50 μl), were performed; the T1 and T2, 24 hours apart and T3-T6 each 48–72 hours.

In one of the three independent experiments, mice were also i.p. injected with BrdU (50 mg/Kg) 24 hours before the AAV-treatment T3-T6.

Tumor size and body weight were measured every two days. Twenty-four hours after the last viral particles injection (pT6) all mice previously euthanized with i.p. injections of tiletamine/zolazepam (800 mg/kg) and xylazine (100 mg/kg), were sacrificed and tumors were harvested, measured, photographed, and pathologically examined. Tumors were then divided in two aliquots, one was fresh frozen (for RNA extraction and qRT-PCR) and the other was fixed for immunohistochemistry analyses.

### Immunohistochemistry and microscopy analysis

Immunohistochemistry (IHC) was performed essentially as previously described [50]. Briefly, excised tumors were fixed with 3.7% paraformaldehyde (PFA) (wt/vol) in PBS for 20 min at room temperature and then embedded in paraffin. Serial sections 5 μm (NeuN) or 8 μm (Ki67 and NeuroD1) thick were cut from the paraffin embedded tissue blocks and floated onto charged glass slides (Super-Frost Plus, Fisher Scientific, Pittsburgh, PA) and dried overnight at 60°C. Sections were deparaffinized in changes of xylene and rehydrated in decreasing concentrations of ethanol and rinsed in PBS. For antigen retrieval, samples were boiled for 20 min in 10 mM sodium citrate buffer (pH 6.0) and cooled for 5–10 min in water. Slides were washed in PBS and incubated with blocking buffer (1x PBS, 0.1% Triton X-100, 1% BSA, 4% donkey serum) for 1 hour and then incubated for 16 hours at 4°C with primary antibodies diluted in blocking buffer. The following day slides were washed three times with washing buffer (1x PBS, 0.1% Triton X-100), incubated with biotinylated anti-Ig secondary antibody for 5 hours followed by streptavidin Alexa 488, and finally washed as before and mounted using Mowiol 4–88 mounting media.

The detection of the BrdU incorporation was performed as previously described [38, 43]. Briefly, the tumors of mice i.p. injected with BrdU were fixed by transcardiac perfusion with 4% PFA in PBS and then cryoprotected in 30% sucrose in PBS. The tumor tissues were embedded in Tissue-Tek OCT (Sakura Finetek, Torrance, CA, USA) and were cut on a rotary microtome in one-in-six series free-floating sections 40 μm thick. BrdU incorporation was detected by treating the sections with 2 N HCl 45 min at 37°C, followed by 0.1 M sodium borate buffer, pH 8.5, for 10 min. The sections were incubated 16 hours at 4°C with a rat monoclonal antibody against BrdU together with the primary antibodies against Ki67 and NeuroD1, then with the appropriate diluted secondary antibodies.

Nuclei were stained using Hoechst 33258 (1 mg/ml; Sigma-Aldrich). Negative controls were obtained by omitting primary antibodies.

Images (20–25 images/each nodule for Ki67, NeuroD1 and BrdU staining) were collected either with an Olympus AX70 fluorescence microscope using 40x lens served by an Olympus XM10 camera and processed using the Olympus CellSens Standard 1.8.1 software or a TCS SP5 laser scanning confocal microscope (Leica Microsystems, Wetzlar, Germany). Labeled cells were counted with the ImageJ software (National Institutes of Health, USA) or with the I.A.S. software (Delta Sistemi, Rome, Italy). The NeuN immunolabelled paraffin sections were analyzed with an Olympus FV1200 laser scanning confocal microscope using a PlanApoN 60x Oil SC (NA = 1.40) Olympus objectives, with optical pinhole at 1AU and a multiline argon (405 nm, 488 nm) laser as excitation source. Confocal Z-stacks were collected at 0.5 μm intervals in a 3.5 μm total optical depth. Approximately 40 representative images from two different paraffin sections, at 30 μm of distance, were acquired for further analysis with ImageJ. Images for direct comparison were collected under same parameters upon setting on negative sample and representative images were chosen.

Images of free-floating sections (40 μm) of nodules injected with AAV-*GFP* virus stained with Hoechst 33258 were collected with a TCS SP5 laser scanning confocal microscope (Leica Microsystems) using a 63x lens and analyzed for the presence of the GFP fluorescent protein.

### RNA extraction, Real time qPCR

Total cellular RNA was extracted from nodules treated with AAV-*Tis21* or with AAV-CBA (control), or from DAOY and D283 human cells infected with AAV-*Tis21* or with AAV-CBA as reported above, and reverse-transcribed as previously described [51]. Three nodules per group and 3–5 technical replicates for the human cell lines were used for analysis. Real-time qPCR was carried out with a 7900HT System (Applied Biosystems) using SYBR Green I dye chemistry in duplicate samples. Relative quantification was performed by the comparative cycle threshold method [52]. The mRNA expression values were normalized to those of the *TATA-binding protein* gene, used as endogenous control. Specific qRT-PCR primers used were deduced from published murine and human cDNA sequences and are listed in Table 1.

**Table 1:** Primer list for qRT-PCR.

Gene	Primer Sequence (5’–3’)
**Mouse Cyclin D1**	**F^a^: AGCCCAACCGAGACCACAG** **R^b^: TCTCCACTTCGCAGCACAGG**
**Mouse Cyclin E**	**F: GTCCTCGCTGCTTCTGCTTTG** **R: CACCACTGATAACCTGAGACCTTC**
**Mouse Cyclin B1**	**F: TGTCTTGCTTGGCTTCATTCATAGTAG** **R: CCACACCCATCTGGCTGTCAG**
**Mouse Cyclin A2**	**F: GCAGCCAGACATCACTAACAGC** **R: CCACCTCAACCAGCCAGTCC**
**Mouse NeuroD1**	**F: GGGTTATGAGATCGTCACTA** **R: ATGTTTCCACTTCCTGTTG**
**Mouse Id3**	**F: CCCAGCCCTCTTCACTTACCC** **R: ACCCAAGTTCAGTCCTTCTCTCG**
**Mouse Tis21**	**F: ATGAGCCACGGGAAGAGAACC** **R: CTCAGGAGACTGGAGAGGAAACC**
**Mouse cdc6**	**F: TCACATGTCTGCAGGGAAGG** **R: CCACACAAACTTGTATGATGCCA**
**Human Cyclin D1**	**F: CGATGCCAACCTCCTCAACG** **R: GGTCTCTCCGCCTTCAGC**
**Human TBP**	**F: GGTGGTGTTGTGAGAAGATGGATG** **R: CGGTGGGCACTTACAGAAGGG**
**Mouse TBP**	**F: CCAATGACTCCTATGACCCCTA** **R: CAGCCAAGATTCACGGTAGAT**

aF, forward.

bR, reverse.

### Statistical analysis

All statistical tests were performed using GraphPad Prism (GraphPad Software, La Jolla, CA, USA) or StatView 5.0 software (SAS Institute, Cary, NC, USA).

For the tumor growth analysis the data are presented as the variance of multiple independent experiments (mean ± SEM). Significance was determined using a two-way ANOVA test followed by post-hoc Bonferroni’s multiple comparison test between the experimental and control samples. The immunohistochemistry data, being percent values whose distribution may not have equal variance, were analyzed with the non parametric Mann-Whitney U test. qRT-PCR data were analyzed by Student’s *t*-test on RQ values or two-way ANOVA followed by Fisher’s PLSD multiple comparison test in experiments of DAOY and D283 cells. The threshold for statistical significance was set at p-values lower than 0.05 for Student’s *t*-test or for Mann-Whitney U test, and at p-values lower than or equal to 0.0009 for Bonferroni’s test (in order to correct for FWE [FamilyWise Error]).

## Results and discussion

Our aim was to evaluate the effectiveness of a gene therapy approach using Tis21, by testing whether the AAV-mediated overexpression of *Tis21* in neoplastic GCPs is able to suppress proliferation and induce their terminal differentiation. Therefore, we established MB allografts of neoplastic GCPs generated from *Ptch1*^+/-^ mice, and developed a gene therapy protocol with AAV particles carrying *Tis21*.

### Establishment of MB allograft

It has been recently shown that grafts of MB cancer cell lines in nude mice are less predictive than MB primary tumors. In fact, the Shh pathway activity, normally very high in tumors derived from *Ptch1*^+/-^ mice, is suppressed in MB explanted from *Ptch1*^+/-^ mice and grown in culture and in MB cancer cell lines [49]. Indeed, MB cancer cell lines have been shown to be negative for the Shh effector *Gli1* gene [49]. For this reason, we have analyzed the MB suppressor activity of *Tis21* using allografts of MB obtained from *Ptch1*^+/-^ mice.

We selected primary tumors with no obvious ulceration or necrosis, because both factors reduce the recovery of viable cells. Single cell suspensions were prepared using collagenase type IV and hyaluronidase from MB tumor with a typical yield of 2–4 x 107 cells per tumor as described in Materials and methods. Next, *Foxn1nu/Foxn1*+ female mice were subcutaneously grafted with 2.5–3 x 106 cells with matrigel. We achieved 100% allograft growth when we injected a suspension containing at least 1.8 x 106 MB cells. Injection of more than 2.5 x 106 cells accelerated the appearance of detectable tumors of 100–150 mm3 (1 week for 2.5 x 106 *vs* 2 weeks for 1.8 x 106 cells).

### AAV-*Tis21* infects MB cell lines and blocks MB allograft growth during treatment

Since the MB primary tumor cells grow well in nude mice, they provide a model for the study of novel gene therapy protocols. As a proof of principle we tested the effects of *Tis21* overexpression, known to be an important tumor suppressor gene and potential relevant target gene in MB treatment. To this aim, we have used an AAV vector that has proven to be neural specific [46, 53], kindly provided to us by Dr. Klein. The efficiency and specificity of the virus is increased by the presence in the backbone of the AAV of the woodchuck hepatitis virus post-transcriptional regulatory element (WPRE) and a cytomegalovirus/chicken β-actin (CBA) promoter, previously shown to drive more efficiently than other promoters gene expression in different brain structures when stereotaxically injected [53, 54]. An important feature of this vector is that the CBA promoter drives gene expression not only in adult neurons, but also in proliferating neural precursors [55] such as neoplastic GCPs. The choice of AAV as carrier was motivated by the fact that this virus is the most used in gene therapy procedures for its high expression and lack of adverse immunological reactions [46].

**Fig 1:**
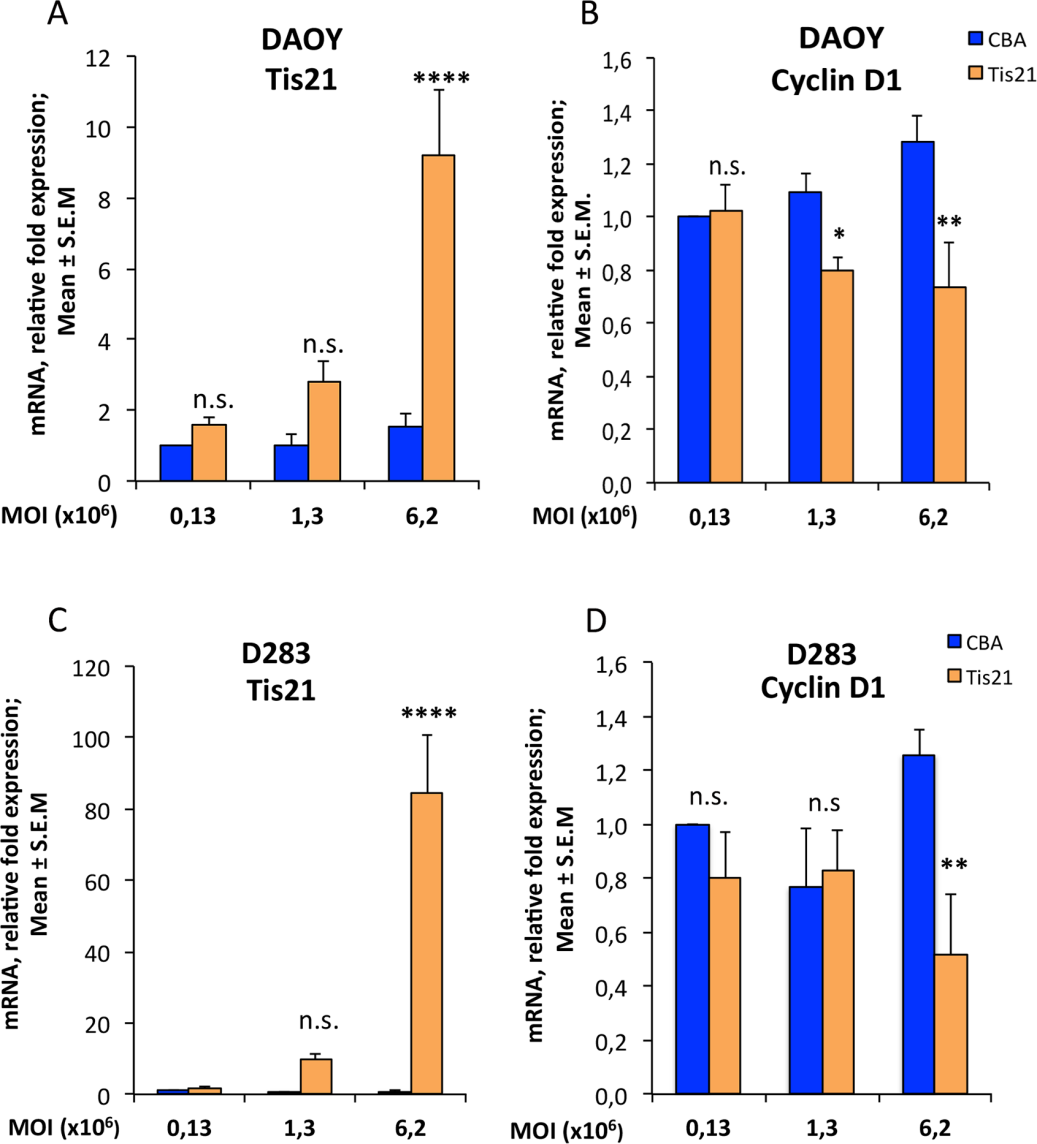
AAV-*Tis21* infects DAOY and D283 MB cell lines and decreases *Cyclin D1* mRNA expression levels in *Tis21* dose-dependent manner. Real-time PCR analysis of mRNA obtained from DAOY (A and B) and D283 (C and D) cells infected with AAV overexpressing *Tis21* (AAV-*Tis21*) or AAV-CBA (control). Increasing MOI employed for infection is indicated in graphs. Average ± SEM values are from at least three independent qPCR experiments and are shown as fold change relative to the control sample (cells infected with the MOI 0.13x106 of AAV-CBA), which was set to unit. Human *TATA-binding protein* mRNA was used as endogenous control for normalization. Two-way ANOVA followed by Fisher's PLSD test was used for the statistical analysis; * p < 0.05, ** p < 0.01, or **** p < 0.0001, n.s. p non-significant, PLSD ANOVA test.

As a preliminary step, we tested the efficiency of the AAV-*Tis21* and of the control AAV-CBA viruses to infect medulloblastoma cell lines and to inhibit a proliferation marker such as cyclin D1, a known target of Tis21 [51]. Two human cell lines, widely used in *in vitro* studies and in allograft experiments, were selected: DAOY derived from the desmoplastic human medulloblastoma subtype [56] and D283 established from malignant ascites cells and a peritoneal medulloblastoma metastasis [57]. The cells were transduced with increasing amount of viral particles (MOI: 0.13x106, 1.3x106, 6.2x106) and collected after 7 days for RNA extraction and for subsequent analyses. In parallel, cells were transduced with the same amount of the empty virus AAV-CBA, as control (Fig 1).

As the MOI of AAV-*Tis21* increased, a parallel increase in levels of *Tis21* mRNA expression was observed in both cell lines, with respect to control treated cells, indicating that AAV-*Tis21* is suitable to infect cells and overexpress *Tis21* (Fig 1A, DAOY MOI 0.13x106: p = 0.613; MOI 1.3x106: p = 0.1391; MOI 6.2x106: 449% increase, p < 0.0001; Fig 1C, D283 MOI 0.13x106: p = 0.932; MOI 1.3x106: p = 0.365; MOI 6.2x106: 100-fold increase, p < 0.0001). It is worth noting that the primers used cross-react also with the human *Tis21* and thus a basal level of the endogenous gene was detected.

As a consequence of the *Tis21* overexpression, it was observed a significant dose-dependent decrease of *cyclin D1* mRNA levels both in DAOY cells (Fig 1B, MOI 0.13x106: p = 0.8664; MOI 1.3x106: 26% decrease, p = 0.0347; MOI 6.2x106: 42.7% decrease, p = 0.001) and in D283 cells (Fig 1D, MOI 0.13x106: p = 0.4074; MOI 1.3x106: p = 0.8095; MOI 6.2x106: 59% decrease, p = 0.0078). We conclude that AAV-*Tis21* infects efficiently human medulloblastoma cell lines and has an inhibitory effect on *cyclin D1*, and can therefore be used to attempt the inhibition of the growth of tumor allografts.

As a next step, we tested the specificity of infection *in vivo* by analyzing MB nodules of nude mice injected with AAV-*GFP* viral particles. As shown in S1 Fig, we observed GFP positive nodule cells indicating that the AAV is able to infect the cells of the MB nodule.

**Fig 2:**
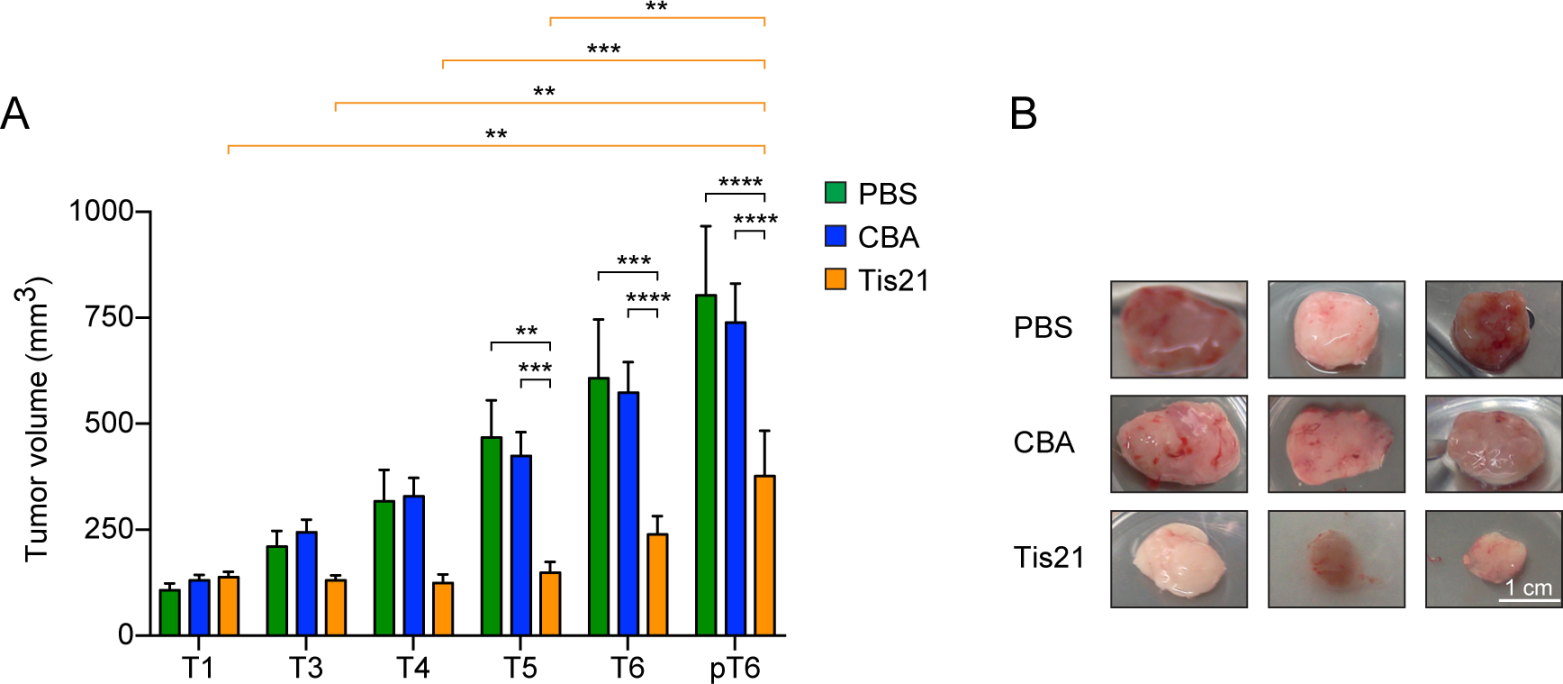
Inhibition of *Ptch1*^+/-^ MB growth in an allograft murine model by *Tis21* overexpression. A) Tumor volumes of nodules obtained by grafting primary *Ptch1*^+/-^ MB tumor cells subcutaneously in nude mice flanks. The bars indicate the average ± SEM of the tumor volume at the time of the viral particles injection (T1-T6) and 24 hours after the last treatment (pT6) with PBS (green), AAV-*Tis21* (orange) and AAV-CBA (blue). Groups of 3–5 mice treated with AAV-*Tis21* and AAV-CBA in three independent experiments and groups of 2 mice treated with PBS in two independent experiments were analyzed. A two-way ANOVA followed by post-hoc Bonferroni's multiple comparison test was used for the statistical analysis; ** p < 0.01, *** p < 0.001, **** p < 0.0001. B) Representative images of AAV-CBA, PBS and AAV-*Tis21*-treated nodules are shown. Scale bars: 10 mm.

In order to evaluate the effect of *Tis21*, groups of 2–5 mice were injected in the allograft with AAV-*Tis21* and control AAV-CBA viral particles, or with PBS (blank control) as described in Materials and methods. We found that AAV-*Tis21* treatment drastically reduces the *Ptch1*^+/-^ derived MB growth. In particular the tumor growth was completely blocked during the first injections with AAV-*Tis21* (T1-T5) and the nodules size was significantly smaller than the control ones 24 hour after the last injection (pT6) (Fig 2). On the contrary, control MB allografts treated with either AAV-CBA or with PBS showed, as expected, an exponential tumor growth (Fig 2; two-way ANOVA, treatment F[2,120] = 27.84, p < 0.0001; single comparisons by Bonferroni's test: AAV-CBA-T5 or PBS-T5 *vs* AAV-*Tis21*-T5 p = 0.0007 or 0.0038, respectively; AAV-CBA-T6 or PBS-T6 *vs* AAV-*Tis21*-T6 p < 0.0001 or 0.0003, respectively; AAV-CBA-pT6 or PBS-pT6 *vs* AAV-*Tis21*-pT6 p < 0.0001 for both). However, a strong growth inhibitory effect of AAV-*Tis21* was observed only during treatment, indeed the *Tis21*-treated MB allograft reach a tumor volume similar to the pT6 MB control in 1–2 weeks after the last viral particles injection (AAV-*Tis21* 653.33 ± 86.7 mm3 *vs* AAV-CBA 611.00 ± 86.0 mm3, n = 3 per group, p > 0.05).

We also checked whether the effect observed on nodule growth corresponded to an increase of *Tis21* expression. To this aim, we tested the expression of *Tis21* mRNA by *in situ* hybridization, using a probe that detects endogenous as well as exogenous *Tis21* mRNA (S2 Fig). We observed that in general the expression of *Tis21* mRNA is higher in AAV-*Tis21* infected nodules; moreover, the expression of *Tis21* signal within the nodule is not uniform, likely depending on the sites of injection of the virus.

### AAV-*Tis21* treatment decreases the number of proliferating cells and increases the number of differentiated cells in MB allograft

**Fig 3:**
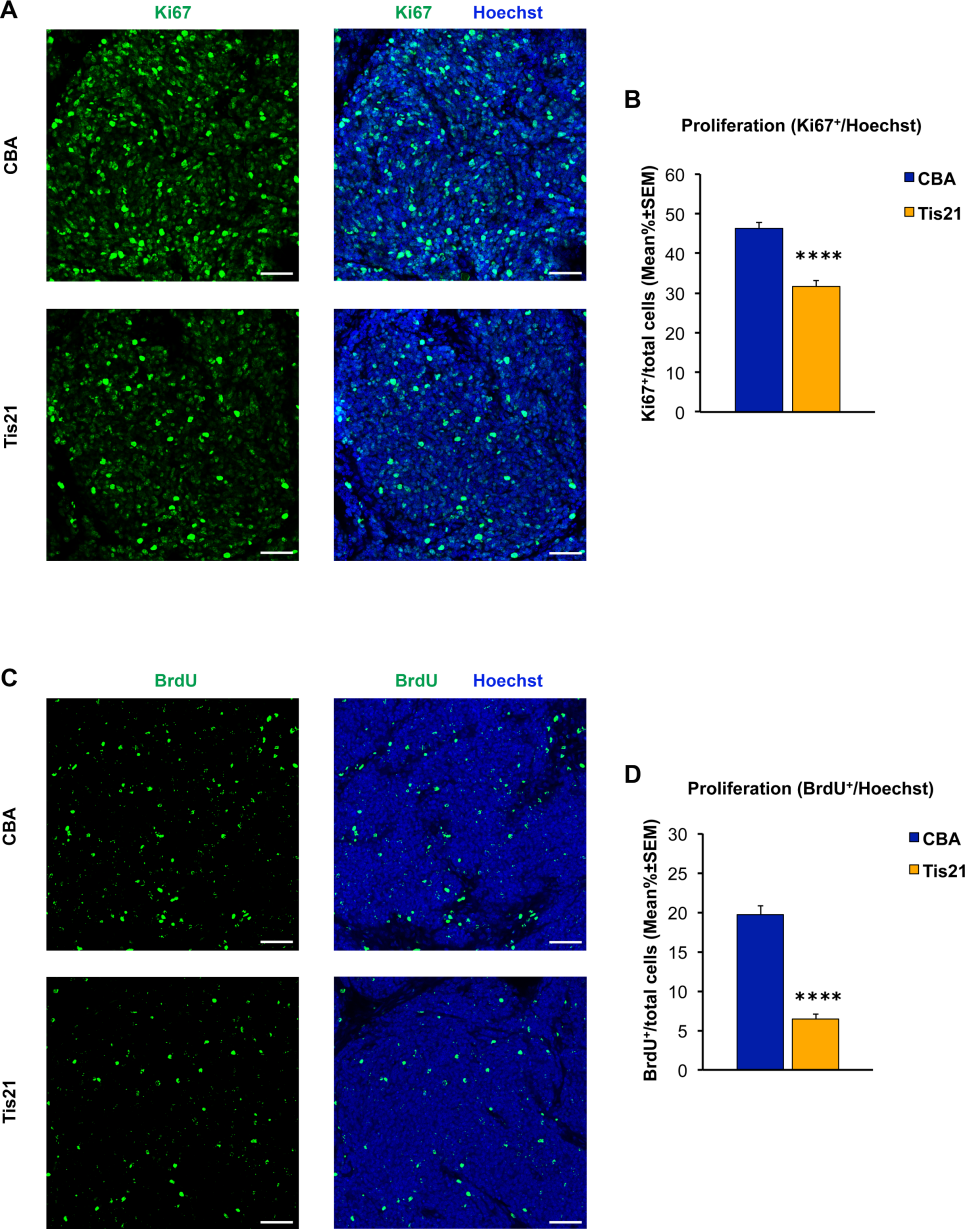
Decreased numbers of cells positive for Ki67 and BrdU in nodules of MB allograft treated with AAV-*Tis21* viral particles. A) Representative confocal images of IHC staining for the proliferation marker Ki67 (green) and for the nuclei dye Hoechst (blue). Scale bars: 50 μm. B) Quantification of the percentage ratio of cells positive for the proliferation marker Ki67 to the total number of cells (Hoechst-positive) in nodules of MB allograft treated with AAV-*Tis21* (orange) and AAV-CBA (blue). **** p < 0.0001, Mann-Whitney U test; mice analyzed: n = 7 for AAV-CBA, n = 8 for AAV-*Tis21*; fields analyzed: n = 207 for AAV-CBA, n = 241 for AAV-*Tis21*. C) Representative confocal images of cells that have entered the cell cycle S-phase, either of AAV-*Tis21*-treated or of AAV-CBA-treated MB allograft, identified as BrdU+ cells (green). Sections are counterstained with Hoechst 33258 to visualize the nuclei. Scale bars: 50 μm. D) Quantification of proliferating cells, measured as mean ± SEM percentage ratio between number of BrdU+ cells and total number of cells (Hoechst-positive) in nodules of MB allograft treated with AAV-*Tis21* (orange) and AAV-CBA (blue). **** p < 0.0001, Mann-Whitney U test; mice analyzed: n = 4 for AAV-CBA, n = 4 for AAV-*Tis21*; fields analyzed: n = 144 for AAV-CBA, n = 144 for AAV-*Tis21*.

Immunohistochemical analysis of MB allograft slides with Ki67 antibody confirmed a statistically significant decrease of cell proliferation in the AAV-*Tis21* treated-allograft (pT6, 24 hours post-treatment; 32% decrease; p < 0.0001) when compared with control allograft samples (Fig 3A and 3B). The proliferation rate was also examined in MB allograft sections of nude mice treated with four i.p. injection of BrdU, which is incorporated in the DNA of the cells entering S-phase. The percentage of BrdU-positive proliferating cells also was found to be decreased significantly (68% decrease; p < 0.0001) when compared with AAV-CBA-treated control mice (Fig 3C and 3D).

**Fig 4:**
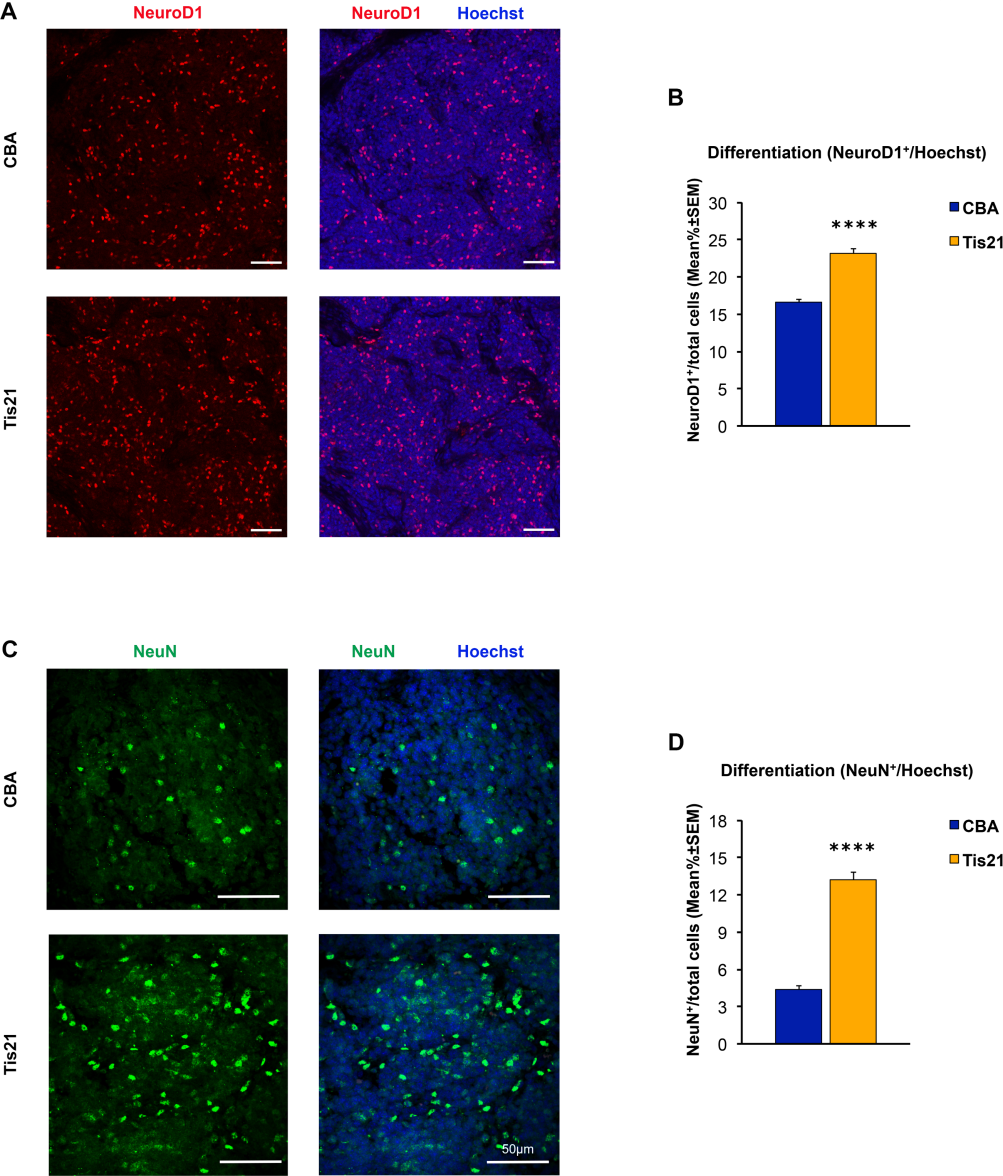
Increased numbers of cells positive for NeuroD1 and NeuN in nodules of MB allograft treated with AAV-*Tis21* viral particles. A) Representative images of IHC staining for the differentiation marker NeuroD1 (red) and for the nuclei dye Hoechst (blue). Scale bars: 50 μm. B) Quantification of the percentage ratio of cells positive for NeuroD1 in nodules of MB allograft treated with AAV-*Tis21* (orange) and AAV-CBA (blue), to the total number of cells (Hoechst-positive). **** p < 0.0001, Mann-Whitney U test. Mice analyzed: n = 7 for AAV-CBA, n = 7 for AAV-*Tis21*; fields analyzed: n = 201 for AAV-CBA, n = 207 for AAV-*Tis21*. C) Representative images of IHC staining for the differentiation marker NeuN (green). In blue the nuclei. Scale bars: 50 μm. D) Quantification of the percentage of NeuN positive cells to the total number of cells (Hoechst-positive). Orange bar (AAV-*Tis21*), blue bar (AAV-CBA). **** p < 0.0001, Mann-Whitney U test. Mice analyzed: n = 2 for AAV-CBA, n = 3 for AAV-*Tis21*; fields analyzed: n = 31 for AAV-CBA, n = 41 for AAV-*Tis21*.

Tis21 has been previously shown to induce cell differentiation in the developing cerebellum [36], as well as in the adult neurogenic niches of the dentate gyrus of the hippocampus [58–60] and of the subventricular zone [61]. Thus, immunohistochemical analysis was performed with NeuroD1 and NeuN antibodies as markers of early and late neuronal differentiation, respectively [62, 63]. As shown in Fig 4A and 4B, the percentage of the NeuroD1-positive cells in the AAV-*Tis21*-treated MB allograft increases of about 39% *vs* control AAV-CBA-treated tumors (i.e., from 16.5% in AAV-CBA to 23% in AAV-*Tis21*; p < 0.0001). Interestingly, also the number of NeuN positive cells is significantly increased in the experimental allograft when compared with the control samples (about three-fold increase, from 4.3% in AAV-CBA to 13.2% in AAV-*Tis21*, p < 0.0001; Fig 4C and 4D). Therefore, importantly, Tis21 induces also the differentiation of MB cells in the subcutaneous allograft (Fig 4).

Overall, these results suggest that *Tis21* overexpression in MB nodules reduces tumor growth via both antiproliferative and pro-differentiative effects.

### AAV-*Tis21* treatment modulates the expression of Tis21 target genes in MB allograft tissues

It is known that Tis21 acts as transcriptional co-regulator and that it controls transcriptional expression of several target genes, involved both in cell cycle and in neuronal differentiation. In particular, *cyclin D1* mRNA levels decrease subsequent to *Tis21* overexpression in different cell lines and tissues [51]. A similar effect is exerted by Tis21 on the *Id3* gene, inhibitor of neuronal differentiation [59]. Thus, we thought to analyze by qRT-PCR the mRNA levels of some known Tis21 target genes and those of *cyclins* and *NeuroD1* to investigate whether: i) the effect on proliferation exerted by Tis21 require the inhibition of *cyclins* (in particular of *cyclin D1*); and ii) the effect on neuronal differentiation occurs via *Id3* regulation.

**Fig 5:**
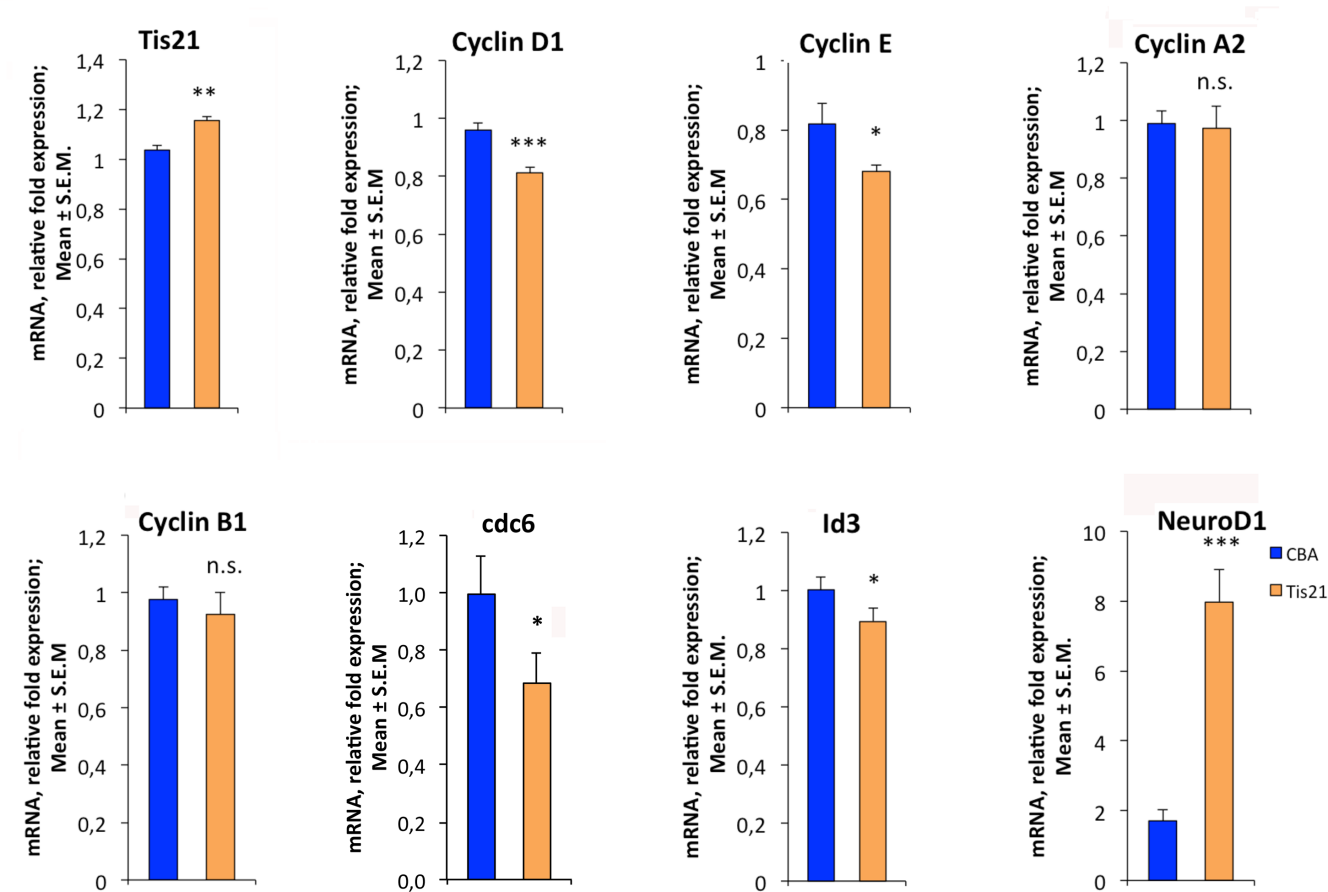
AAV-*Tis21* treatment decreases *Cyclin D1* and *Cyclin E* and increases *NeuroD1* mRNA expression levels in MB allograft tissues. Real-time PCR analysis of RNA obtained from the nodules treated with AAV overexpressing *Tis21* (AAV-*Tis21*) or AAV-CBA (control). Three nodules per group were analyzed. Average ± SEM values are from (at least) three independent experiments and are shown as fold change relative to the control sample (one nodule treated with AAV-CBA), which was set to unit. *TATA-binding protein* mRNA was used as endogenous control for normalization. * p < 0.05, ** p < 0.01, or *** p < 0.001, n.s. p non-significant, Student’s *t* -test.

To this aim we analyzed by qRT-PCR 3 AAV-*Tis21* and 3 AAV-CBA control nodules and observed a small but significant increase (11.9%, p = 0.0024) of *Tis21* mRNA expression in AAV-*Tis21* nodules relative to the AAV-CBA nodules (Fig 5). The weak increase, albeit significant, of *Tis21* expression could be explained by the fact that the *Tis21* signal within the nodule is not uniform, as observed by *in situ* hybridization (S2 Fig), thus yielding a low level of *Tis21* mRNA detected by qRT-PCR within the whole nodule. Moreover, we cannot exclude a low mRNA stability in highly proliferating cells. However, the increased expression of *Tis21* obtained with this gene therapy protocol was sufficient to cause a statistically significant decrease of *cyclin D1* (15.4%, p = 0.00024) and *cyclin E* (16.7%, p = 0.049) mRNA levels with respect to control (Fig 5). No significant differences were observed for *cyclin A2* and *cyclin B1* expression levels (p = 0.85 and p = 0.061, respectively), suggesting that Tis21 influences the G1/S transition phase of cell cycle in this model system. As a further attempt to test the contribution of Tis21 on the G1/S transition we analyzed the expression of *cdc6*. This is a constituent of the replication initiation machinery and represents a marker of cell proliferation [64], overexpressed in different types of human cancers including medulloblastoma [65]. We observed that *cdc6* mRNA levels are significantly reduced by Tis21 (31% decrease, p = 0.046; Fig 5). Finally, we recorded a statistically significant reduction of *Id3* (10.8%, p = 0.044) and a significant increase of *NeuroD1* (366%, p = 0.00022) mRNA levels.

It is worth noting that even a small change in *cyclin D1* levels is sufficient to cause great changes in proliferation. In fact, our previous data in cerebellum showed that a limited but selective increase of *cyclin D1* levels is associated to changes in GCPs proliferation [44]. Overall, these results demonstrate that Tis21 has a double action able to reduce proliferation and induce differentiation of primary MB cells by regulating key target genes.

## Conclusion

Our study demonstrated, in a model of MB allograft in nude mice, that overexpression of *Tis21* by AAV strongly impairs tumor growth under treatment pressure, and delays tumor growth in the short post-treatment time. The effect on tumor cell growth is likely to be due to both a decrease in cell proliferation (Ki67 and BrdU markers) and an increase of cell differentiation (NeuroD1 and NeuN markers). Moreover, the antiproliferative action exerted by *Tis21* overexpression could be due to an effect on key cell cycle genes. In particular, as expected, Tis21 acts by inhibiting the cyclins that control the G1/S transition phase of the cell cycle. Moreover, *Tis21* overexpression clearly induces an increase of *NeuroD1* mRNA (about four-fold increase) and, in parallel, of NeuroD1-positive and NeuN-positive cells. The therapeutic relevance of this effect lies in the fact that tumor cells that have differentiated leave permanently the tumor program.

Our data suggest the possibility that the pro-differentiative action of Tis21, documented by the increase of NeuroD1 and NeuN expression in allografts, could be mediated by down-regulation of *Id3* mRNA levels, as we previously reported in the analysis of dentate gyrus [59, 60]. Of course it seems as well possible that the cyclin-dependent antiproliferative action of Tis21 contributes to the exit from cell cycle of tumor cells and ultimately to their differentiation.

Overall the results confirm the role of *Tis21* as a MB suppressor gene and validate *Tis21* as a potential relevant target for gene therapy in brain tumors. Furthermore, we observe that even with a limited expression of AAV in tumor cells *in vivo*, a large inhibitory effect on tumor growth is obtained. Further studies may aim to improve gene therapy protocols in terms of efficiency and duration of *Tis21* expression.

## Supporting information

S1 Fig AAV efficiently infects MB nodule cells. Representative confocal images of sections from nodules infected with AAV-*GFP* or AAV-CBA viruses and stained for the nuclei dye Hoechst (blue). Green fluorescence indicates GFP-positive cells. Scale bars: 50 μm.
(TIF)

S2 Fig AAV-*Tis21* infects MB nodule cells and expresses exogenous *Tis21*. Representative sections (4x magnification) of two nodules infected with AAV-CBA (A, A’) and two nodules infected with AAV-*Tis21* (B, B’), showing the expression of *Tis21* mRNA labeled by *in situ* hybridization. *Tis21* mRNA expression is higher in AAV-*Tis21* infected nodules (see enlargements 20x of boxed areas). Scale bars: 500 μm and 100 μm (enlargements).
(TIF)

S1 File Supplemental materials and methods. *In situ* Hybridization.
(DOCX)
