# Supplementary figures and images for: Tis21-gene therapy inhibits medulloblastoma growth in a murine allograft model
Source: PLoS One. 2018 Mar 14;13(3):e0194206. doi: 10.1371/journal.pone.0194206 (PMC5851620; doi:10.1371/journal.pone.0194206)

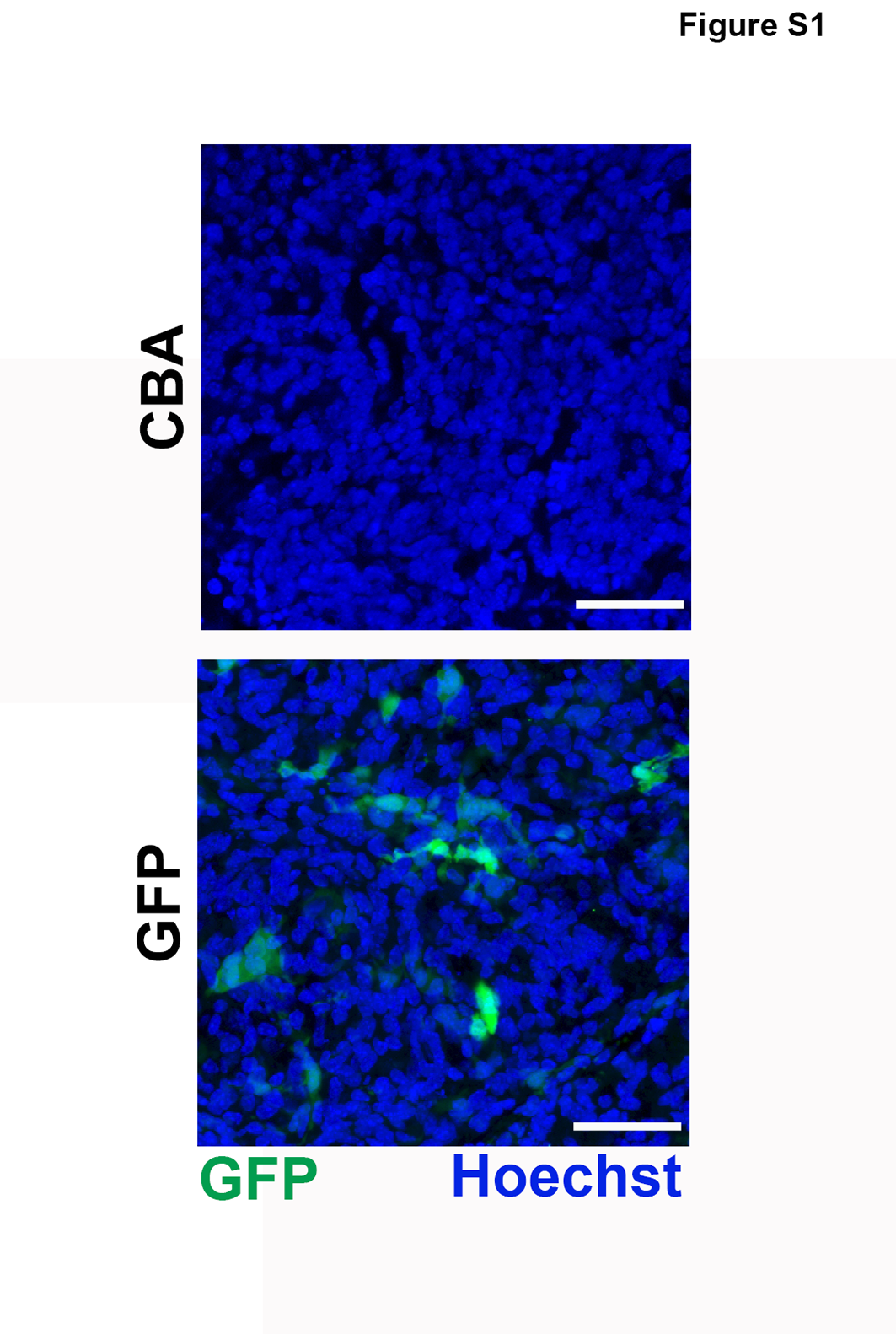

Supplement: S1 Fig — Representative confocal images of sections from nodules infected with AAV-GFP or AAV-CBA viruses and stained for the nuclei dye Hoechst (blue). Green fluorescence indicates GFP-positive cells. Scale bars: 50 μm. (TIF) [file pone.0194206.s001.tif]

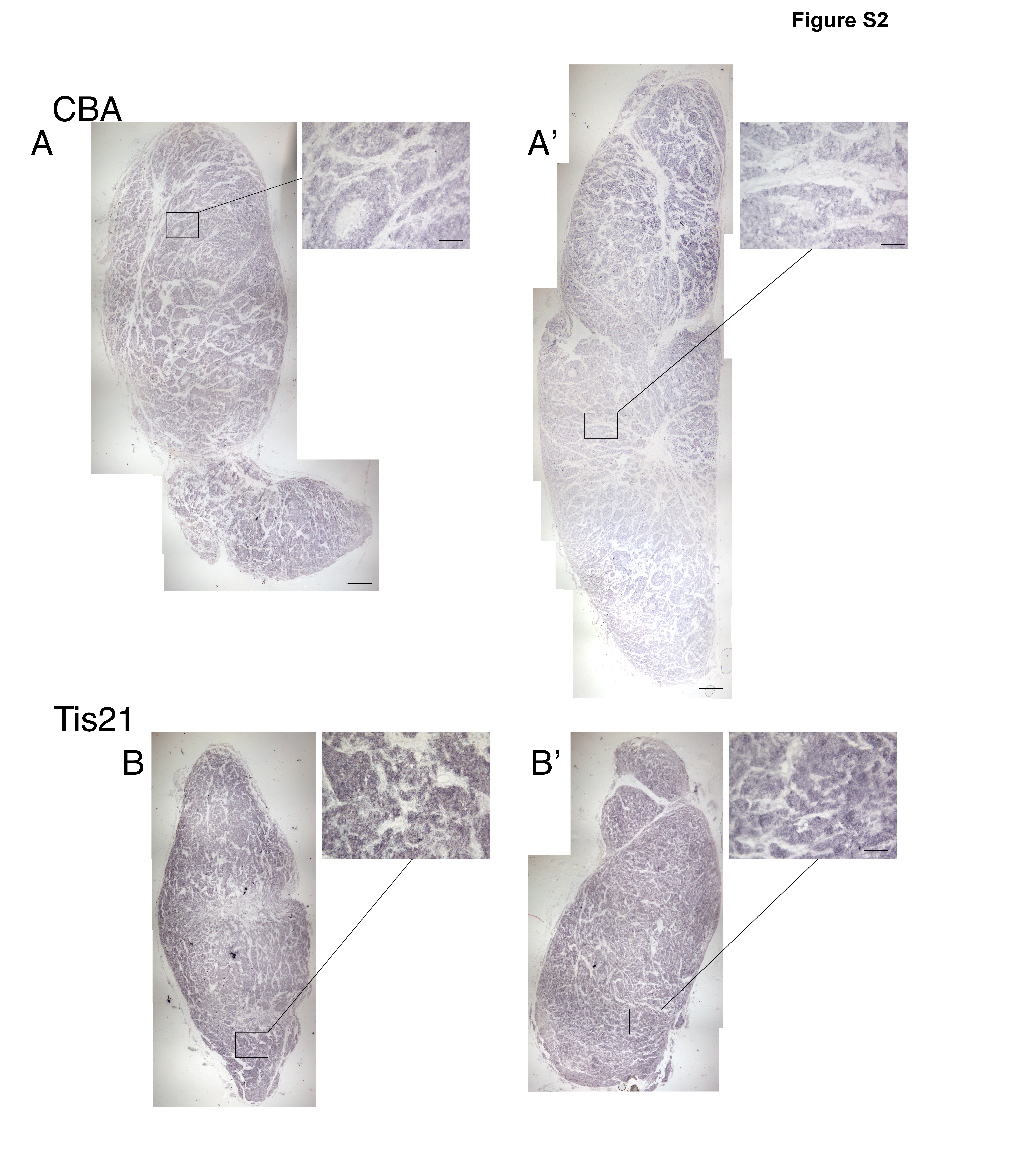

Supplement: S2 Fig — Representative sections (4x magnification) of two nodules infected with AAV-CBA (A, A’) and two nodules infected with AAV-Tis21 (B, B’), showing the expression of Tis21 mRNA labeled by in situ hybridization. Tis21 mRNA expression is higher in AAV-Tis21 infected nodules (see enlargements 20x of boxed areas). Scale bars: 500 μm and 100 μm (enlargements). (TIF) [file pone.0194206.s002.tif]
