# Supplementary material for: Tis21-gene therapy inhibits medulloblastoma growth in a murine allograft model
Source: PLoS One. 2018 Mar 14;13(3):e0194206. doi: 10.1371/journal.pone.0194206 (PMC5851620; doi:10.1371/journal.pone.0194206)
Supplement: S1 File — In situ Hybridization. (DOCX) [file pone.0194206.s003.docx]

# Supplemental Materials and methods

# *In situ* Hybridization

Excised nodules were fixed and cut (8 μm sections) as described in “Immunohistochemistry and microscopy analysis” section. The hybridization was performed as reported previously [36]. Antisense probes detecting mouse *Tis21* mRNA were transcribed by T7 polymerase using as template the pEX-A128 vector (synthesized by Genscript, Piscataway, NJ, USA) that carried the *Tis21* ORF region in the XbaI 5’-HindIII 3’ sites.

The riboprobe was labeled with digoxigenin-UTP (Transcription kit; Roche Products, Basel, Switzerland), following the protocol of the manufacturer. No signal was detected by the sense probe.

Hybridization was performed at 60°C for 18 hr. Samples were incubated overnight at 4°C with alkaline phosphatase-conjugated anti-digoxigenin antibody (1:2000; Roche Products), washed, and processed for colometric detection using 5-bromo-4-chlor-indolyl-phosphate/nitroblue–tetrazolium–chloride.
